# Dr. Charles Ernest Baker

**Published:** 1909-04-03

**Authors:** 


Dr. Charles Ernest Baker last week died suddenly in London at the age of forty-four. He was a Fellow of the Royal College of Surgeons and a Licentiate of the Royal College of Physicians, and he was for four years clinical assistant at the Royal Eye Hospital, Southwark. Dr. Baker had also held appointments at the Royal Orthopædic Hospital, the Royal Free Hospital, St. Bartholomew's Hospital, and the East London Hospital for Children.

